# Enhanced Antioxidant and Anti-Inflammatory Activities of *Diospyros lotus* Leaf Extract via Enzymatic Conversion of Rutin to Isoquercitrin

**DOI:** 10.3390/antiox14080950

**Published:** 2025-08-02

**Authors:** Yeong-Su Kim, Chae Sun Na, Kyung-Chul Shin

**Affiliations:** 1 R&D Center, Ildusanbang Co., Cheongsong 37434, Republic of Korea; kansok2@gmail.com; 2 Department of Wild Plants and Seeds Conservation, Baekdudaegan National Arboretum, Bonghwa 36209, Republic of Korea; 3 Department of Bioscience and Biotechnology, Hankuk University of Foreign Studies, Yongin 17035, Republic of Korea

**Keywords:** *Diospyros lotus*, isoquercitrin, rutin, α-l-rhamnosidase, antioxidant, anti-inflammatory, enzymatic bioconversion

## Abstract

Isoquercitrin, a monoglucoside form of quercetin, exhibits superior antioxidant, anti-inflammatory, and cardiovascular protective effects in comparison to its precursor, rutin. However, its natural abundance is limited. This study aimed to increase the functional value of *Diospyros lotus* leaf extract through enzymatic conversion of rutin to isoquercitrin using α-l-rhamnosidase and to evaluate the changes in biological activities after conversion. A sugar-free *D. lotus* leaf extract was prepared and subjected to enzymatic hydrolysis with α-l-rhamnosidase under optimized conditions (pH 5.5, 55 °C, and 0.6 U/mL). Isoquercitrin production was monitored via high-performance liquid chromatography. Antioxidant and anti-inflammatory activities were assessed using the 2,2-diphenyl-1-picrylhydrazyl radical scavenging and lipoxygenase (LOX) inhibition assays, respectively. The enzymatic reaction resulted in complete conversion of 30 mM rutin into isoquercitrin within 180 min, increasing isoquercitrin content from 9.8 to 39.8 mM. The enzyme-converted extract exhibited significantly enhanced antioxidant activity, with a 48% improvement in IC_50_ value compared with the untreated extract. Similarly, LOX inhibition increased from 39.2% to 48.3% after enzymatic conversion. Both extracts showed higher inhibition than isoquercitrin alone, indicating synergistic effects of other phytochemicals present in the extract. This study is the first to demonstrate that α-l-rhamnosidase-mediated conversion of rutin to isoquercitrin in *D. lotus* leaf extract significantly improves its antioxidant and anti-inflammatory activities. The enzymatically enhanced extract shows potential as a functional food or therapeutic ingredient.

## 1. Introduction

*Diospyros lotus*, commonly known as date-plum, is a deciduous tree belonging to the Ebenaceae family that has long been used in traditional medicine in East Asia [1,2]. Its leaves contain abundant polyphenolic compounds, particularly flavonoids such as rutin, which contribute to its antioxidant and anti-inflammatory effects [1,3,4]. However, the full pharmacological potential of *D. lotus* leaf extract remains unrealized due to the limited bioavailability of its major constituents.

Flavonoids, which are polyphenolic secondary metabolites widely distributed in plants, are known for their diverse health-promoting effects, including antioxidant, anti-inflammatory, and cardiovascular protection [5,6,7]. Among them, quercetin is one of the most well-studied flavonols; however, its low water solubility and poor oral bioavailability limit its therapeutic application [8,9,10,11]. To overcome these limitations, quercetin glycosides such as rutin (quercetin-3-*O*-rutinoside) and isoquercitrin have been explored as alternatives that exhibit improved pharmacokinetics [12,13].

Rutin is composed of a quercetin core linked to a disaccharide (rhamnose and glucose), whereas isoquercitrin contains only a glucose moiety. This structural difference significantly affects their absorption and metabolism [14,15]. Rutin requires microbial hydrolysis in the colon, resulting in low and delayed quercetin release, while isoquercitrin is readily absorbed and efficiently converted into quercetin in vivo [15,16,17]. Due to its superior bioavailability, isoquercitrin exhibits stronger antioxidant and anti-inflammatory effects than both rutin and quercetin [18,19]. It effectively scavenges reactive oxygen species and inhibits inflammatory cytokines such as TNF-α and IL-6 [20,21,22,23]. Moreover, it shows benefits in cardiovascular protection, glucose metabolism, and allergic disease management [24,25,26].

Given these advantages, isoquercitrin is considered a superior alternative to rutin and quercetin. However, its low natural abundance has drawn interest in its production from rutin-rich plant sources such as *D. lotus* via enzymatic conversion. Enzymatic bioconversion offers a promising strategy to enhance the functional quality of plant extracts by converting glycosylated flavonoids into more bioactive forms. In particular, various studies have utilized almond-derived β-glucosidase, snailase, and β-glucosidase from *Pyrococcus furiosus* to hydrolyze glucose moieties from flavonoid glucosides in plant extracts such as citrus peel, *Sophora japonica*, and buckwheat, thereby enhancing their bioavailability and biological activity [27,28,29,30]. α-l-Rhamnosidase (EC 3.2.1.40) is a glycoside hydrolase that cleaves terminal α-l-rhamnose residues and enables the conversion of rutin to isoquercitrin [31,32,33,34,35,36]. While such transformations have been previously studied using purified substrates, direct enzymatic modification of complex plant matrices such as *D. lotus* extract has rarely been reported.

*D. lotus* was chosen as the plant material for this study due to its high rutin content and long-standing use in traditional medicine. This research aimed to apply α-l-rhamnosidase in the conversion of rutin into isoquercitrin in *D. lotus* leaf extract and investigate the effects of this conversion on the extract’s biological activities. The optimized enzymatic reaction significantly increased isoquercitrin content, and the antioxidant and anti-inflammatory properties of the enzyme-converted extract were evaluated and compared with those of the untreated extract. To the best of our knowledge, this is the first report to demonstrate enhanced functionality of *D. lotus* extract through α-l-rhamnosidase-mediated enzymatic conversion.

## 2. Materials and Methods

### 2.1. Materials

The flavonoid standards rutin, quercetin-3-*O*-neohesperidoside, kaempferol 3-*O*-rutinoside, kaempferol-3-*O*-neohesperidoside, narirutin, naringin, hesperidin, neohesperidin, isoquercitrin, kaempferol 3-*O*-glucoside, prunin, and hesperetin-7-*O*-glucoside were purchased from Chemfaces (Wuhan, China) and Sigma-Aldrich (St. Louis, MO, USA). Commercial α-l-rhamnosidase was purchased from Megazyme (Bray, Ireland). *D. lotus* leaves were collected from plants grown at Namhu-Myen, Andong-Si, Kyungsangbuk-do, Republic of Korea on 19 September 2023 and identified by Dr. Chae Sun Na (Baekedudaegan National Arboritum, Republic of Korea). The collected leaves were dried for a week at 15 °C, 15% relative humidity. The dried leaves were then sealed in an aluminum bag with silica gel and stored at −20 °C until the experiment.

### 2.2. Preparation of D. lotus Leaf Extract

A crude extract containing flavonoids such as rutin and isoquercitrin was prepared by treating 100 g of dried *D. lotus* leaf powder with 1 L of 95% ethanol. The mixture was subjected to ultrasonic extraction trice at 45 °C for 3 h each. After cooling, insoluble materials were removed via filtration through a 0.45 μm membrane. Ethanol was evaporated, and the residue was dissolved in 1 L of distilled water. The aqueous solution was applied to a glass column (700 mm × 41.4 mm) packed with Diaion HP-20 resin (Mitsubishi Chemical Corporation, Tokyo, Japan) to eliminate free sugars, which are known to interfere with enzymatic activity. Washing with 2 L of distilled water was performed to remove unbound hydrophilic substances, while flavonoid-containing compounds remained adsorbed on the resin. These retained compounds were subsequently eluted using 2 L of ethanol at a flow rate of 0.5 mL/min. Following evaporation of ethanol, the eluate was reconstituted in 1 L of distilled water to obtain a sugar-free extract. This extract was adjusted to a final concentration of 10% (*w*/*v*) and used as the substrate for enzymatic conversion of rutin into isoquercitrin by α-l-rhamnosidase.

### 2.3. Hydrolytic Activity

Enzymatic activity was expressed in units (U), where one unit is defined as the amount of enzyme required to produce 1 µmol of isoquercitrin from rutin per minute at pH 6.0 and 55 °C. Substrate specificity toward flavonoids was assessed by conducting the reaction at 50 °C for 30 min in 50 mM McIlvaine buffer (pH 6.0) containing 1 mM flavonoids, 0.005 U/mL α-l-rhamnosidase, and 5% dimethylsulfoxide (DMSO). In this reaction system, DMSO served to enhance the solubility of flavonoid substrates and products. Specific activity (U/mg) was defined as the amount of flavonoid products, including isoquercitrin, kaempferol 3-*O*-glucoside, prunin, and hesperetin-7-*O*-glucoside, produced per milligram of enzyme per minute under the defined reaction conditions.

### 2.4. Environmental Conditions

The effects of pH and temperature on the hydrolytic activity of α-l-rhamnosidase were evaluated using *D. lotus* leaf extract containing 20 mM rutin as the substrate. The pH was adjusted from 4.0 to 8.0 at a fixed temperature of 50 °C, while reaction temperature was varied from 30 to 70 °C at pH 5.5. Reactions were performed for 30 min in 50 mM McIlvaine buffer containing the leaf extract, 0.2 U/mL enzyme, and 5% (*v*/*v*) DMSO.

For thermal stability analysis, the enzyme solution was incubated in 50 mM McIlvaine buffer (pH 5.5) at five different temperatures (45, 50, 55, 60, and 65 °C). Enzymatic activity was measured at various time intervals. The thermal inactivation kinetics followed a first-order model, described by the equation ln(E_t_/E_0_) = −k_d_t, where E_0_ and E_t_ represent the initial and residual enzyme activity at time t, respectively. The deactivation rate constant (k_d_, min^−1^) was obtained from the slope of the linear plot, and the thermal half-life (t_1/2_) was calculated as t_1/2_ = ln(2)/k_d_.

### 2.5. Enzymatic Conversion of Rutin to Isoquercitrin in D. lotus Leaf Extract

Optimal enzyme concentrations for converting rutin to isoquercitrin in *D. lotus* leaf extract were determined by varying the concentration of α-l-rhamnosidase in the range of 0.1 to 1 U/mL. The reaction mixture consisted of 50 mM McIlvaine buffer (pH 5.5), *D. lotus* leaf extract standardized to contain 20 mM rutin, and 5% (*v*/*v*) DMSO and was incubated at 55 °C for 120 min. To identify the optimal substrate concentration, rutin levels within the extract were adjusted by varying the extract concentration from 5 to 40 mM, and 0.6 U/mL of α-l-rhamnosidase was used under the same reaction conditions. Time-course analysis of isoquercitrin production was carried out using *D. lotus* leaf extract containing 30 mM rutin with 0.6 U/mL of enzyme. Reactions were conducted in 50 mM McIlvaine buffer (pH 5.5) with 5% DMSO at 55 °C and monitored for up to 180 min.

### 2.6. DPPH Assay

A 2,2-diphenyl-1-picrylhydrazyl (DPPH) radical scavenging assay was conducted using a 0.2 mM DPPH solution prepared in methanol. In a 96-well microplate, 150 μL of the DPPH solution was mixed with 50 μL of *D. lotus* leaf extract or its biotransformed form at final concentrations of 15.6, 31.3, 62.5, 125, 250, and 500 μg/mL or with 50 μL of methanol as the blank. The reaction mixtures were incubated at 25 °C for 30 min, after which absorbance was measured at 520 nm using a microplate reader. Radical scavenging activity was calculated using the following equation: (*A*_control_ *− A*_sample_)/*A*_control_ × 100, where *A*_control_ is the absorbance value of the DPPH solution with methanol and *A*_sample_ is the absorbance value in the presence of the extract. Ascorbic acid in aqueous solution was included as a reference antioxidant. The half-maximal inhibitory concentration (IC_50_) values were calculated from the dose-response curves using nonlinear regression analysis. In addition, the area under the curve (AUC) was determined by applying the trapezoidal rule to the scavenging activity plotted against extract concentration. The resulting AUC values are expressed as %·μg/mL, representing the integrated radical scavenging activity (% inhibition) over the concentration range (μg/mL).

### 2.7. Lipoxygenase Assay

The inhibitory effects of isoquercitrin on lipoxygenase (LOX) activity were evaluated using a LOX inhibitor screening assay kit (Cayman Chemical, Ann Arbor, MI, USA) at a final concentration of 50 μM for test samples and reference compounds. For test samples, the *D. lotus* leaf extract was prepared to contain a total of 50 μM of flavonoids, consisting of rutin and isoquercitrin, while the enzyme-converted extract contained 50 μM of isoquercitrin only. Nordihydroguaiaretic acid (NDGA) and baicalein (5,6,7-trihydroxyflavone) were used as positive controls for LOX inhibition and anti-inflammatory activity, respectively. The assay was performed by adding 10 μL of methanol and 90 μL of 15-LOX enzyme to each well of a 96-well microplate, followed by the addition of 10 μL of arachidonic acid to initiate the enzymatic reaction. After shaking the plate for 5 min, 100 μL of chromogenic reagent was added to terminate the reaction. The formation of hydroperoxides, resulting from the LOX-mediated oxidation of arachidonic acid, was quantified by measuring absorbance at 500 nm. The percentage of LOX inhibition was calculated using the following formula: (C − T)/C × 100, where C and T represent the absorbance values in the absence and presence of the test sample, respectively.

### 2.8. High-Performance Liquid Chromatography Analysis

An equal volume of n-butanol was added to the reaction mixture to stop the enzymatic reaction and to extract the substrates and products. After phase separation, the n-butanol and aqueous layers were obtained, and the organic fraction was collected. The n-butanol layer was evaporated to dryness, and the residue was then redissolved in methanol. The resulting solution was analyzed via high-performance liquid chromatography using an Agilent 1260 system (Santa Clara, CA, USA) equipped with a UV detector at 205 nm and a reversed-phase AccQ-Tag column (150 × 3.9 mm; Waters, Milford, MA, USA). Chromatographic separation was performed at 30 °C using a gradient elution of solvent I (0.1% trifluoroacetic acid in acetonitrile, *v*/*v*) and solvent II (0.1% trifluoroacetic acid in distilled water, *v*/*v*). The elution profile comprised a gradient from 10:90 to 40:60 over 40 min at a flow rate of 1.0 mL/min, followed by a shift from 40:60 to 90:10 over the next 5 min.

The substrates rutin, quercetin-3-*O*-neohesperidoside, kaempferol 3-*O*-rutinoside, kaempferol-3-*O*-neohesperidoside, narirutin, naringin, hesperidin, and neohesperidin were detected at retention times of 7.3, 6.5, 8.5, 7.8, 8.2, 8.9, 9.6, and 10.4 min, respectively. Their corresponding products, namely isoquercitrin, kaempferol 3-*O*-glucoside, prunin, and hesperetin-7-*O*-glucoside, were eluted at 7.7, 9.6, 8.5, and 10.1 min, respectively. Each compound was identified and quantified by comparing its retention time and peak area with those of authentic flavonoid standards, using external calibration at 205 nm.

### 2.9. Statistical Analysis

All experiments were performed in triplicate (*n* = 3), and results are presented as mean ± standard deviation (SD). Statistical comparisons of biological activity data were conducted using one-way ANOVA followed by Tukey’s HSD post hoc test, with *p* < 0.05 considered statistically significant. All analyses were performed using SigmaPlot version 12.0 (Systat Software, San Jose, CA, USA).

## 3. Results and Discussion

### 3.1. Substrate Specificity of α-l-Rhamnosidase for Flavonoids

Although the substrate specificity of α-l-rhamnosidase was previously characterized for several rhamnose-containing flavonoids, including rutin, hesperidin, and naringin [37], its specificity for a broader set of structurally diverse substrates has not been fully explored.

**Figure 1:**
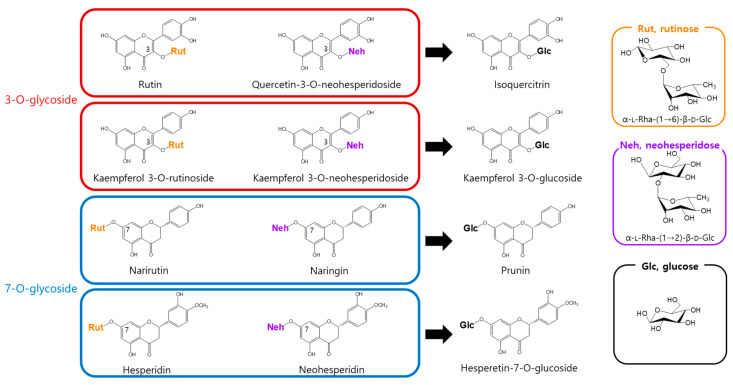
Conversion of rhamnose-containing flavonoids by α-l-rhamnosidase.

To address this, we assessed the enzymatic activity of α-l-rhamnosidase toward eight flavonoid glycosides bearing either rutinose [α-l-rhamnopyranosyl-(1→6)-β-d-glucopyranose] or neohesperidose [α-l-rhamnopyranosyl-(1→2)-β-d-glucopyranose]. α-l-Rhamnosidase catalyzed the hydrolysis of the terminal rhamnose moiety in all tested flavonoids, producing the corresponding mono-*O*-glucoside derivatives (Figure 1).

α-l-Rhamnosidase exhibited higher activity toward rutinose-containing flavonoids (narirutin, hesperidin, rutin, and kaempferol-3-*O*-rutinoside) than toward neohesperidose-containing ones (naringin, neohesperidin, quercetin-3-*O*-neohesperidoside, and kaempferol-3-*O*-neohesperidoside). Among the rutinose-type substrates, compounds with a 3-*O*-glycosidic linkage, namely rutin and kaempferol-3-*O*-rutinoside, exhibited higher activity than those with a 7-*O*-glycosidic linkage, namely narirutin and hesperidin. In contrast, in the neohesperidose group, higher activity was observed for 7-*O*-glycosides (naringin and neohesperidin) than for 3-*O*-glycosides (quercetin-3-*O*-neohesperidoside and kaempferol-3-*O*-neohesperidoside; Table 1). Notably, α-l-rhamnosidases purified from *Bacteroides* JY and *Pseudomonas paucimobilis* have been shown to prefer neohesperidose-containing flavonoids such as naringin over rutinose-containing substrates like hesperidin or rutin [38,39]. In contrast, the enzyme purified from *Fusobacterium* K-6 displayed higher activity toward hesperidin than naringin but showed markedly lower activity toward rutin, with approximately 4.8-fold less activity than that for naringin [40].

Among all the tested substrates, α-l-rhamnosidase exhibited the highest activity toward rutin. Therefore, rutin was selected as the target substrate for subsequent experiments. In particular, *D. lotus* leaf extract, which naturally contains rutin, was used for the optimization of the enzymatic conversion into isoquercitrin under various reaction conditions.

### 3.2. Effects of pH and Temperature on the Production of Isoquercitrin from Rutin in D. lotus Leaf Extract

The contents of rutin and isoquercitrin in the dried *D. lotus* leaf material were 2.57 mg/g and 0.64 mg/g, respectively. No other major flavonoid components were detected under the applied analytical conditions, and thus only rutin and isoquercitrin were quantified. Furthermore, no conversion was observed in the absence of enzyme under any tested condition, confirming that the transformation was strictly enzyme-dependent.

**Figure 2:**
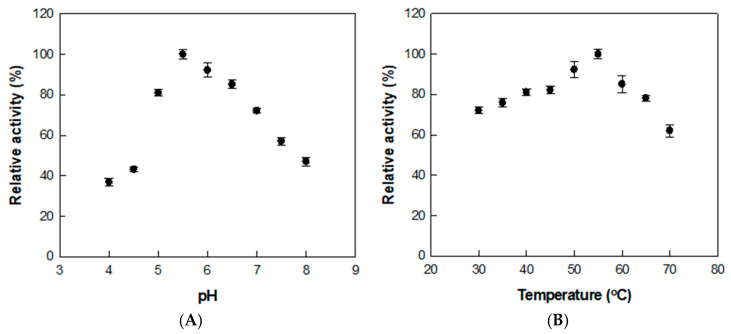
Effects of (**A**) pH and (**B**) temperature on α-l-rhamnosidase activity for *D. lotus* leaf extract. Data represent the means of three experiments, and error bars represent the standard deviation.

The conversion of rutin to isoquercitrin was optimized by treating the extract with α-l-rhamnosidase under varying pH and temperature conditions. The enzyme activity was highest at pH 5.5 and 55 °C, as shown by assays conducted across pH 4.0–8.0 and temperatures ranging from 30 to 70 °C (Figure 2), where relative activities in each panel were normalized independently.

Previous studies using reagent-grade rutin as the substrate reported an optimal pH and temperature of 6.0 and 50 °C, respectively [37]. The slight shift in both optimal pH and temperature is presumed to result from the complex nature of the *D. lotus* extract. The presence of organic acids, flavonoids, and metal ions may influence the enzyme’s structural conformation, charge distribution, or substrate binding, thereby altering its activity profile under extract-based conditions.

**Figure 3:**
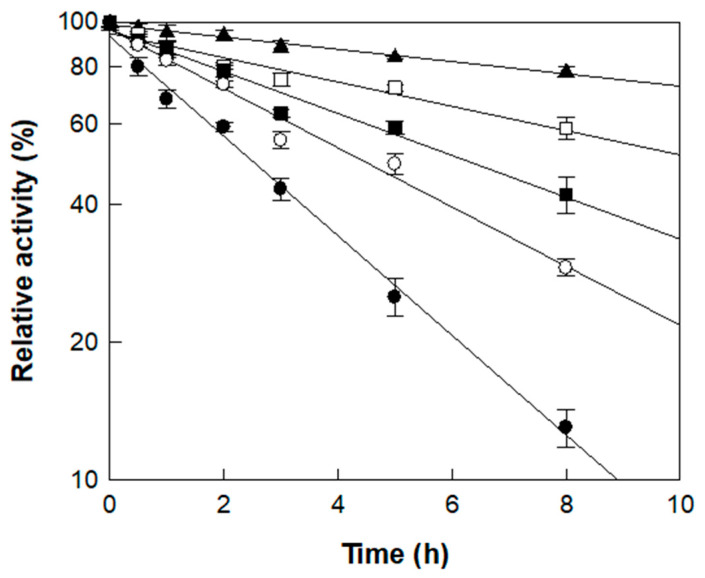
Thermal stability of α-l-rhamnosidase at different temperatures. Symbols (▲, □, ■, ○, ●) correspond to incubation temperatures of 45, 50, 55, 60, and 65 °C, respectively, measured across multiple time points. Enzyme samples were collected at designated intervals, and residual activity is expressed as a percentage relative to the initial activity (set as 100%). Data represent the means of three experiments, and error bars represent the standard deviation.

The thermal stability of α-l-rhamnosidase was investigated across temperatures from 45 to 65 °C using rutin in *D. lotus* extract as the substrate (Figure 3). The enzyme retained a half-life of 22.5 h at 45 °C, which progressively decreased to 11.3 h at 50 °C, 6.6 h at 55 °C, 4.7 h at 60 °C, and 2.8 h at 65 °C. The deactivation rate constant (k_d_) at 45 °C and 50 °C was approximately 3.5- and 1.7-fold lower than that at 55 °C, respectively, indicating higher stability at lower temperatures. In contrast, the enzyme was inactivated more rapidly at higher temperatures, with k_d_ values being 1.4- and 2.0-fold higher at 60 and 65 °C, respectively, than at 55 °C (Table 2).

Notably, the half-lives determined in this study were considerably longer than those reported in a previous study using reagent-grade rutin (7.0 h at 50 °C and 4.1 h at 60 °C) [37], suggesting that certain components of *D. lotus* extract exert a protective effect on the enzyme during thermal exposure. Based on catalytic performance and thermal stability, 55 °C was deemed suitable for the enzymatic conversion of rutin to isoquercitrin in *D. lotus* leaf extract. As the reaction was completed within 3 h, as will be shown in the time-course study below, the enzyme’s half-life of 6.6 h at 55 °C was sufficient to support the process, making this temperature appropriate as the optimal condition.

### 3.3. Effects of Enzyme and Substrate Concentrations on the Production of Isoquercitrin from Rutin in D. lotus Leaf Extract

**Figure 4:**
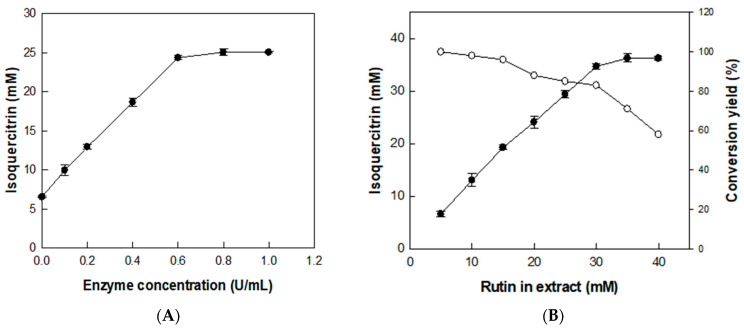
Effects of (**A**) enzyme and (**B**) substrate concentrations on the production of isoquercitrin from rutin in *D. lotus* leaf extract. Data represent the means of three experiments and error bars represent the standard deviation.

The effect of enzyme concentration on the production of isoquercitrin from rutin was investigated using *D. lotus* leaf extract containing 20 mM rutin (with 6.5 mM isoquercitrin) as a substrate and with enzyme concentration varying from 0.1 to 1 U/mL for 2 h (Figure 4A). The isoquercitrin production from rutin in *D. lotus* leaf extract gradually increased with increasing enzyme concentrations. However, increasing the enzyme concentration to >0.6 U/mL decreased the isoquercitrin production rate from rutin, indicating that the optimal enzyme concentration was 0.6 U/mL. Under this enzyme concentration, the effect of substrate concentration was investigated by varying rutin concentrations in *D. lotus* leaf extract from 5 to 40 mM for 2 h (Figure 4B). The converted isoquercitrin increased with increasing concentrations of rutin in *D. lotus* leaf extract to 30 mM, indicating that the optimal substrate concentration for isoquercitrin production was 30 mM rutin in *D. lotus* leaf extract.

### 3.4. Time-Course Reaction for the Production of Isoquercitrin from Rutin in D. lotus Leaf Extract

**Figure 5:**
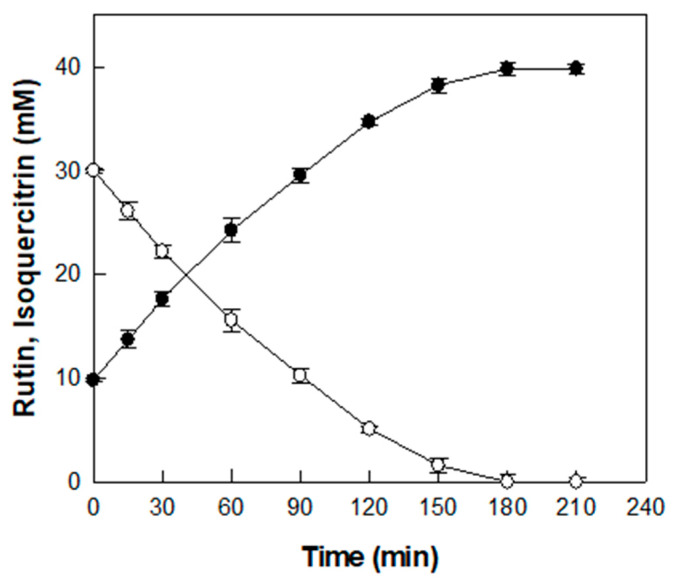
Time-course reaction for the production of isoquercitrin (●) from rutin (○) in *D. lotus* leaf extract. Data represent the means of three experiments, and error bars represent the standard deviation.

The time-course reaction for producing isoquercitrin from rutin in *D. lotus* leaf extract was carried out under the optimized conditions of pH 5.5, 55 °C, 0.6 U/mL α-l-rhamnosidase, and *D. lotus* leaf extract containing 30 mM rutin (Figure 5).

Before enzymatic conversion, an initial concentration of 9.8 mM isoquercitrin was detected in the extract, which is attributed to its native content. As the enzymatic reaction proceeded, isoquercitrin concentration gradually increased and 30 mM rutin was completely converted into isoquercitrin after 180 min with a productivity of 10 mM/h, resulting in a final isoquercitrin concentration of 39.8 mM. Representative HPLC chromatograms at 0, 120, and 180 min are presented in Figure S1. To the best of our knowledge, this study is the first to demonstrate the enzymatic conversion of rutin into isoquercitrin in *D. lotus* leaf extract, which results in a marked increase in isoquercitrin content.

### 3.5. Antioxidant and Anti-Inflammatory Activities of Enzyme-Converted D. lotus Leaf Extract

**Figure 6:**
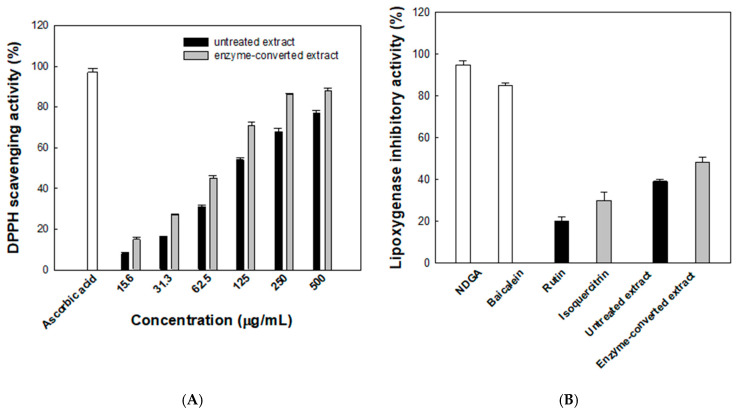
(**A**) Antioxidant activities evaluated using the lipoxygenase 2,2-diphenyl-1-picrylhydrazyl (DPPH) radical scavenging assay. Black and gray bars represent the untreated and enzyme-converted extracts, respectively. (**B**) Anti-inflammatory activities evaluated using the lipoxygenase inhibitory activity assay. Data represent the means of three experiments, and error bars represent the standard deviation. Statistically significant differences among groups were determined by one-way ANOVA followed by Tukey’s post hoc test (*p* < 0.05).

The antioxidant activity of the enzyme-converted *D. lotus* leaf extract was assessed using the DPPH radical scavenging assay. As shown in Figure 6A, both the untreated and enzyme- converted extracts exhibited concentration-dependent increases in radical scavenging activity. At 500 μg/mL, the scavenging activity reached 88% for the enzyme-converted extract and 77% for the untreated extract, compared with 97% for the reference compound, ascorbic acid. Across all tested concentrations, the enzyme-converted extract consistently showed higher radical scavenging activity than the untreated counterpart. Notably, at 125 μg/mL, the enzyme-converted extract achieved 71% scavenging, while the untreated extract showed 54%. The calculated IC_50_ values further support this trend, with the enzyme-converted extract exhibiting a lower IC_50_ of 72.8 μg/mL compared with 107.4 μg/mL for the untreated extract, indicating an improvement in antioxidant efficacy of approximately 48%. This improvement is likely due to the increased isoquercitrin production from rutin by α-l-rhamnosidase. To better evaluate antioxidant efficacy over the entire concentration range, AUC values were calculated. The enzyme-converted extract showed a markedly higher AUC value (36,640%·μg/mL) than the untreated extract (29,328%·μg/mL), indicating a consistently stronger radical scavenging capacity. This integrated parameter complements the IC_50_ analysis and reinforces the enhanced antioxidant efficacy achieved through enzymatic conversion.

The anti-inflammatory potential of the enzyme-converted *D. lotus* leaf extract was assessed by evaluating its inhibitory effect on LOX activity (Figure 6B). NDGA, a well-known LOX inhibitor, and baicalein, a natural anti-inflammatory flavonoid, were used as reference compounds and showed 95% and 85% inhibition, respectively. The enzyme-converted extract showed 48.3% inhibition, which was higher than that for the untreated extract (39.2%). For comparison, the individual flavonoids isoquercitrin and rutin showed 30.6% and 20.1% inhibition, respectively, under the same conditions. Notably, both extracts were adjusted to contain a total of 50 μM flavonoids, with the untreated extract comprising a mixture of rutin and isoquercitrin and the enzyme-converted extract containing primarily isoquercitrin. Despite this, both extracts exhibited greater inhibitory activity than isoquercitrin alone, suggesting that additional phytochemicals present in the *D. lotus* leaf extract contribute synergistically to LOX inhibition.

The enhanced antioxidant and LOX-inhibitory activities observed in the enzyme-converted extract are likely attributable to both the increased isoquercitrin content and the formation of additional bioactive intermediates. Isoquercitrin has been reported to exhibit stronger biological activities than rutin, partly due to its favorable structure that retains free hydroxyl groups while being more readily hydrolyzed in vivo [20,35]. In addition, deglycosylation during enzymatic treatment may generate partially hydrolyzed flavonoids with improved radical scavenging or LOX-inhibitory capacity [41,42]. Since the LOX inhibition assay was conducted using a purified enzyme system, the increased activity reflects direct biochemical interaction rather than membrane-related effects.

## 4. Conclusions

This study demonstrates that enzymatic conversion of rutin to isoquercitrin in *D. lotus* leaf extract using α-l-rhamnosidase significantly enhances its functional properties. The optimized reaction resulted in a marked increase in isoquercitrin content, which correlated with improved antioxidant and anti-inflammatory activities. These effects were greater than those of isoquercitrin alone, suggesting synergistic contributions from other phytochemicals in the extract. Although enzymatic strategies have been applied to other plant extracts, this study presents the first successful application to *D. lotus*, a medicinal plant known for its high rutin content. The broader applicability of this approach to other rutin-rich plant sources may depend on matrix-specific factors such as pH, sugar content, and the presence of inhibitory compounds. In addition, both the *D. lotus* leaf extract and the enzyme used in this study are generally considered safe and commercially available. The enzyme also exhibited favorable thermal stability, suggesting its suitability for cost-effective application in scaled-up processes, particularly when combined with enzyme immobilization or recycling strategies, though further safety evaluation may be required for practical applications. These findings support the use of enzyme-treated *D. lotus* leaf extract as a natural antioxidant ingredient in functional foods, dietary supplements, health beverages, and cosmeceuticals, owing to its enhanced isoquercitrin content.

## Figures and Tables

**Table 1:** Specific activity of α-l-rhamnosidase for flavonoids.

Substrate	Specific Activity (nmol/min/mg)
Rutin	94.04 ± 1.23
Quercetin-3-*O*-neohesperidoside	14.15 ± 2.38
Kaempferol-3-*O*-rutinoside	91.05 ± 0.89
Kaempferol-3-*O*-neohesperidoside	16.35 ± 1.64
Narirutin	71.35 ± 2.19
Naringin	23.51 ± 1.90
Hesperidin	66.10 ± 2.41
Neohesperidin	21.87 ± 0.96

Values are expressed as the mean ± standard deviation (*n* = 3).

**Table 2:** Deactivation constant (k_d_) and half-lives (t_1/2_) of α-l-rhamnosidase at 45, 50, 55, 60, and 65 °C.

Temperature (°C)	k_d_ (h^−1^)	t_1/2_ (h)
45	3.08 × 10^−2^	22.5
50	6.11 × 10^−2^	11.3
55	1.05 × 10^−1^	6.6
60	1.49 × 10^−1^	4.7
65	2.05 × 10^−1^	2.8

## Data Availability

The data presented in this study are available on request from the corresponding author.

## Supplementary Materials

The following supporting information can be downloaded at: https://www.mdpi.com/article/10.3390/antiox14080950/s1, Figure S1: HPLC profiles for the production of isoquercitrin from rutin in *D. lotus* leaf extract.

## References

[1] Uddin G., Rauf A., Siddiqui B.S., Muhammad N., Khan A., Shah S.U. (2014). Anti-nociceptive, anti-inflammatory and sedative activities of the extracts and chemical constituents of *Diospyros lotus* L.. Phytomedicine.

[2] Rauf A., Uddin G., Patel S., Khan A., Halim S.A., Bawazeer S., Ahmad K., Muhammad N., Mubarak M.S. (2017). Diospyros, an under-utilized, multi-purpose plant genus: A review. Biomed. Pharmacother..

[3] Wu H., Zhao W., Zhou J., Xie X., Zhong X., Liu Y., Shi L. (2024). Extraction, analysis of antioxidant activities and structural characteristics of flavonoids in fruits of *Diospyros lotus* L. *LWT*
**2024**, *201*, 116248. LWT.

[4] Gao H., Cheng N., Zhou J., Wang B., Deng J., Cao W. (2014). Antioxidant activities and phenolic compounds of date plum persimmon (*Diospyros lotus* L.) fruits. J. Food Sci. Technol..

[5] Kumar S., Pandey A.K. (2013). Chemistry and biological activities of flavonoids: An overview. Sci. World J..

[6] Middleton E., Kandaswami C., Theoharides T.C. (2000). The effects of plant flavonoids on mammalian cells: Implications for inflammation, heart disease, and cancer. Pharmacol. Rev..

[7] Li M., Qian M., Jiang Q., Tan B., Yin Y., Han X. (2023). Evidence of flavonoids on disease prevention. Antioxidants.

[8] Deepika, Maurya P.K. (2022). Health benefits of quercetin in age-related diseases. Molecules.

[9] Chiang M.C., Tsai T.Y., Wang C.J. (2023). The potential benefits of quercetin for brain health: A review of anti-inflammatory and neuroprotective mechanisms. Int. J. Mol. Sci..

[10] Cai X., Fang Z., Dou J., Yu A., Zhai G. (2013). Bioavailability of quercetin: Problems and promises. Curr. Med. Chem..

[11] Kandemir K., Tomas M., McClements D.J., Capanoglu E. (2022). Recent advances on the improvement of quercetin bioavailability. Trends Food Sci. Technol..

[12] Yin H., Ma J., Han J., Li M., Shang J. (2019). Pharmacokinetic comparison of quercetin, isoquercitrin, and quercetin-3-*O-*β-D-glucuronide in rats by HPLC-MS. PeerJ.

[13] Dehelean C.A., Coricovac D., Pinzaru I., Marcovici I., Macasoi I.G., Semenescu A., Lazar G., Cinta Pinzaru S., Radulov I., Alexa E. (2022). Rutin bioconjugates as potential nutraceutical prodrugs: An in vitro and in ovo toxicological screening. Front. Pharmacol..

[14] Zhang H., Hassan Y.I., Liu R., Mats L., Yang C., Liu C., Tsao R. (2020). Molecular mechanisms underlying the absorption of aglycone and glycosidic flavonoids in a Cac*O*-2 BBe1 cell model. ACS Omega.

[15] Owczarek-Januszkiewicz A., Magiera A., Olszewska M.A. (2022). Enzymatically modified isoquercitrin: Production, metabolism, bioavailability, toxicity, pharmacology, and related molecular mechanisms. Int. J. Mol. Sci..

[16] Ulluwishewa D., Montoya C.A., Mace L., Rettedal E.A., Fraser K., McNabb W.C., Moughan P.J., Roy N.C. (2023). Biotransformation of Rutin in in vitro porcine ileal and colonic fermentation models. J. Agric. Food Chem..

[17] Mbikay M., Chrétien M. (2022). Isoquercetin as an anti-COVID-19 medication: A potential to realize. Front. Pharmacol..

[18] Paulke A., Eckert G.P., Schubert-Zsilavecz M., Wurglics M. (2012). Isoquercitrin provides better bioavailability than quercetin: Comparison of quercetin metabolites in body tissue and brain sections after six days administration of isoquercitrin and quercetin. Pharmazie.

[19] Magalingam K.B., Radhakrishnan A., Haleagrahara N. (2016). Protective effects of quercetin glycosides, rutin, and isoquercetrin against 6-hydroxydopamine (6-OHDA)-induced neurotoxicity in rat pheochromocytoma (PC-12) cells. Int. J. Immunopathol. Pharmacol..

[20] Li X., Jiang Q., Wang T., Liu J., Chen D. (2016). Comparison of the antioxidant effects of quercitrin and isoquercitrin: Understanding the role of the 6″-OH Group. Molecules.

[21] Wang H., Xia W., Long G., Pei Z., Li Y., Wu M., Wang Q., Zhang Y., Jia Z., Chen H. (2020). Isoquercitrin ameliorates cisplatin-induced nephrotoxicity via the inhibition of apoptosis, inflammation, and oxidative stress. Front. Pharmacol..

[22] Li Y., Ma Y., Yao Y., Ru G., Lan C., Li L., Huang T. (2024). Protective effect of isoquercitrin on UVB-induced injury in HaCaT cells and mice skin through anti-inflammatory, antioxidant, and regulation of MAPK and JAK2-STAT3 pathways. Photochem. Photobiol..

[23] Li L., Zhang X.-H., Liu G.-R., Liu C., Dong Y.-M. (2016). Isoquercitrin suppresses the expression of histamine and prO-inflammatory cytokines by inhibiting the activation of MAP Kinases and NF-κB in human KU812 cells. Chin. J. Nat. Med..

[24] Bondonno N.P., Bondonno C.P., Ward N.C., Woodman R.J., Hodgson J.M., Croft K.D. (2020). Enzymatically modified isoquercitrin improves endothelial function in volunteers at risk of cardiovascular disease. Br. J. Nutr..

[25] Chen L., Shen T., Zhang C.P., Xu B.L., Qiu Y.Y., Xie X.Y., Wang Q., Lei T. (2020). Quercetin and isoquercitrin inhibiting hepatic gluconeogenesis through LKB1-AMPKα pathway. Acta Endocrinol..

[26] Rogerio A.P., Kanashiro A., Fontanari C., da Silva E.V., Lucisano-Valim Y.M., Soares E.G., Faccioli L.H. (2007). Anti-inflammatory activity of quercetin and isoquercitrin in experimental murine allergic asthma. Inflamm. Res..

[27] Hostetler G., Riedl K., Cardenas H., Diosa-Toro M., Arango D., Schwartz S., Doseff A.I. (2012). Flavone deglycosylation increases their anti-inflammatory activity and absorption. Mol. Nutr. Food Res..

[28] Kornpointner C., Scheibelreiter J., Halbwirth H. (2022). Snailase: A promising tool for the enzymatic hydrolysis of flavonoid glycosides from plant extracts. Front. Plant Sci..

[29] Slámová K., Kapešová J., Valentová K. (2018). ‘Sweet Flavonoids’: Glycosidase-catalyzed modifications. Int. J. Mol. Sci..

[30] Shin K.C., Nam H.K., Oh D.K. (2013). Hydrolysis of flavanone glycosides by β-glucosidase from *Pyrococcus furiosus* and its application to the production of flavanone aglycones from citrus extracts. J. Agric. Food Chem..

[31] Ge L., Chen A., Pei J., Zhao L., Fang X., Ding G., Wang Z., Xiao W., Tang F. (2017). Enhancing the thermostability of α-L-rhamnosidase from *Aspergillus terreus* and the enzymatic conversion of rutin to isoquercitrin by adding sorbitol. BMC Biotechnol..

[32] Guan C.J., Ji Y.J., Hu J.L., Hu C.N., Yang F., Yang G.E. (2017). Biotransformation of Rutin using crude enzyme from *Rhodopseudomonas palustris*. Curr. Microbiol..

[33] Wang D., Zheng P., Chen P., Wu D. (2020). Highly efficient enzymatic conversion of Rutin to isoquercitrin and l-rhamnose using deep eutectic solvents. ACS Sustain. Chem. Eng..

[34] Li L.J., Liu X.Q., Du X.P., Wu L., Jiang Z.D., Ni H., Li Q.B., Chen F. (2020). Preparation of isoquercitrin by biotransformation of rutin using α-L-rhamnosidase from *Aspergillus niger* JMU-TS528 and HSCCC purification. Prep. Biochem. Biotechnol..

[35] Chen Y., Wang L., Guo Y., Zhang M., Xie H., Xia G., Xu L., Yang H., Shen Y. (2025). Preparation of isoquercitrin and rhamnose from readily accessible rutin by a highly specific recombinant α-L-rhamnosidase (r-Rha1). Nat. Prod. Res..

[36] Shin K.C., Seo M.J., Oh D.K., Choi M.N., Kim D.W., Kim Y.S., Park C.S. (2019). Cloning and characterization of α-L-rhamnosidase from *Chloroflexus aurantiacus* and its application in the production of isoquercitrin from rutin. Biotechnol. Lett..

[37] Kim D.Y., Yeom S.J., Park C.S., Kim Y.S. (2016). Effect of high hydrostatic pressure treatment on isoquercetin production from rutin by commercial α-L-rhamnosidase. Biotechnol. Lett..

[38] Jang J.S., Kim D.H. (1996). Purification and characterization of alpha-L-rhamnosidase from Bacteroides JY-6, a human intestinal bacterium. Biol. Pharm. Bull..

[39] Miake F., Satho T., Takesue H., Yanagida F., Kashige N., Watanabe K. (2000). Purification and characterization of intracellular alpha-L-rhamnosidase from *Pseudomonas paucimobilis* FP2001. Arch Microbiol..

[40] Park S., Kim J., Kim D. (2005). Purification and characterization of quercitrin-hydrolyzing alpha-L-rhamnosidase from *Fusobacterium* K-60, a human intestinal bacterium. J. Microbiol. Biotechnol..

[41] Franco E.P.D., Contesini F.J., Lima da Silva B., Alves de Piloto Fernandes A.M., Wielewski Leme C., Gonçalves Cirino J.P., Bueno Campos P.R., de Oliveira Carvalho P. (2020). Enzyme-assisted modification of flavonoids from *Matricaria chamomilla*: Antioxidant activity and inhibitory effect on digestive enzymes. J. Enzym. Inhib. Med. Chem..

[42] Suh S., Kim Y.E., Yang H.J., Ko S., Hong G.P. (2017). Influence of autoclave treatment and enzymatic hydrolysis on the antioxidant activity of *Opuntia ficus-indica* fruit extract. Food Sci. Biotechnol..

