# Supplementary material for: Enhanced Antioxidant and Anti-Inflammatory Activities of Diospyros lotus Leaf Extract via Enzymatic Conversion of Rutin to Isoquercitrin
Source: Antioxidants (Basel). 2025 Aug 2;14(8):950. doi: 10.3390/antiox14080950 (PMC12382970; doi:10.3390/antiox14080950)
Supplement: Supplementary file 1 [file antioxidants-14-00950-s001.zip › antioxidants-3758436-supplementary.pdf]

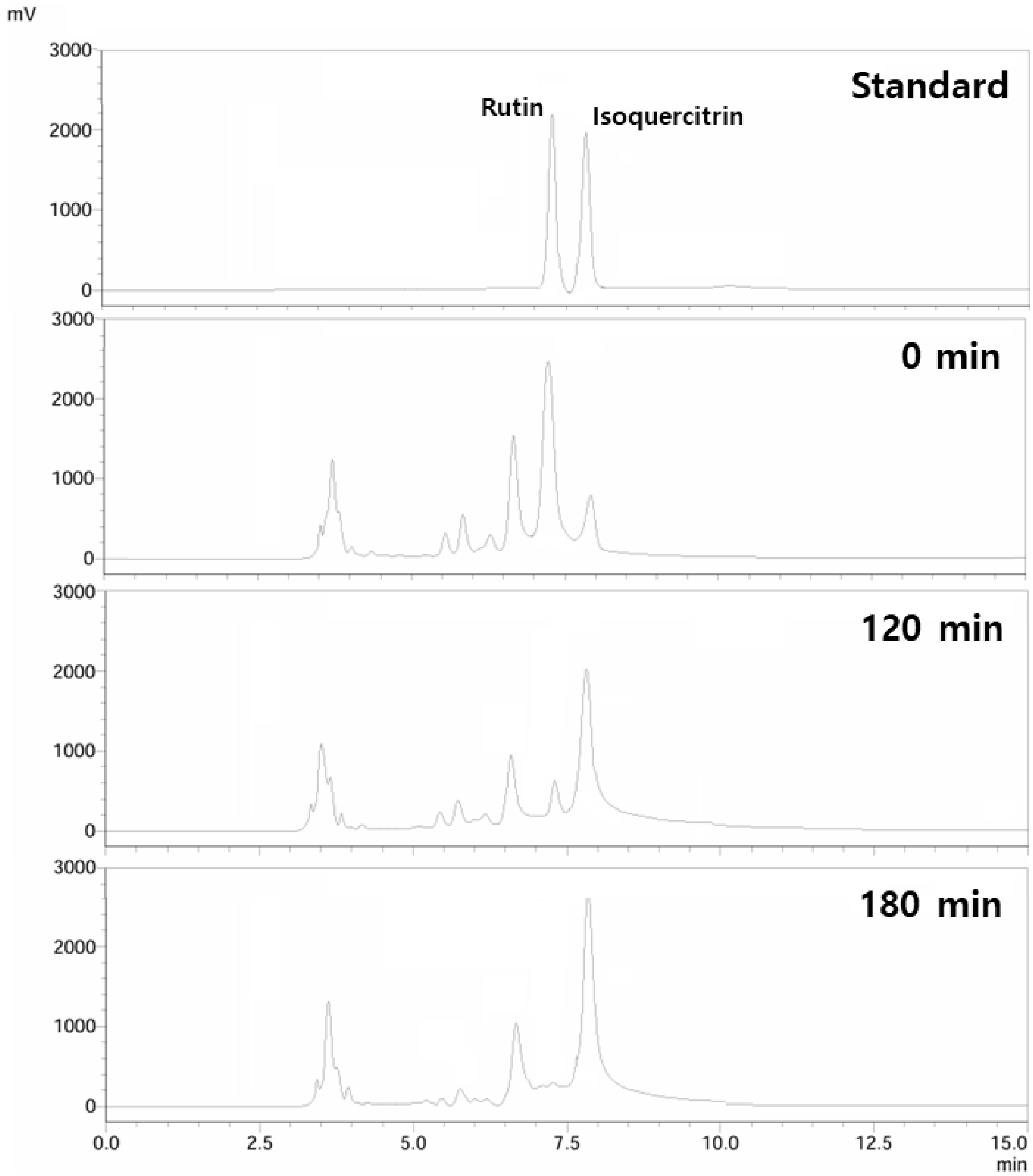

Figure S1. HPLC profiles for the production of isoquercitrin from rutin in *D. lotus* leaf extract. Samples were collected at 0, 120, and 180 min after the addition of α-L-rhamnosidase under optimized conditions. The peak corresponding to rutin (7.3 min) gradually decreased, while the isoquercitrin peak (7.7 min) increased over time.
